# Screen Time, Physical Activity and Self-Esteem in Children: The Ulm Birth Cohort Study

**DOI:** 10.3390/ijerph15061275

**Published:** 2018-06-16

**Authors:** Stefanie Braig, Jon Genuneit, Viola Walter, Stephanie Brandt, Martin Wabitsch, Lutz Goldbeck, Hermann Brenner, Dietrich Rothenbacher

**Affiliations:** 1 Institute of Epidemiology and Medical Biometry, Ulm University; Helmholtzstraße 22, 89081 Ulm, Germany; jon.genuneit@uni-ulm.de (J.G.); dietrich.rothenbacher@uni-ulm.de (D.R.); 2 Division of Clinical Epidemiology and Ageing Research, German Cancer Research Center (DKFZ), Im Neuenheimer Feld 581, 69120 Heidelberg, Germany; v.walter@dkfz-heidelberg.de (V.W.); h.brenner@dkfz-heidelberg.de (H.B.); 3 Division of Pediatric Endocrinology and Diabetes, Department of Pediatrics and Adolescent Medicine, Ulm University, Eythstraße 24, 89075 Ulm, Germany; Stephanie.Brandt@min-ulm.de (S.B.); Martin.Wabitsch@min-ulm.de (M.W.); 4 Department of Child and Adolescent Psychiatry/Psychotherapy, Ulm University, Steinhövelstraße 5, 89075 Ulm, Germany; lutz.goldbeck@min-ulm.de; 5 Division of Preventive Oncology, German Cancer Research Center (DKFZ) and National Center for Tumor Diseases (NCT), Im Neuenheimer Feld 581, 69120 Heidelberg, Germany; 6 German Cancer Consortium (DKTK), German Cancer Research Center (DKFZ), Im Neuenheimer Feld 581, 69120 Heidelberg, Germany

**Keywords:** child, mass media, mental health, self-esteem

## Abstract

Screen time is a central activity of children’s daily life and jeopardizes mental health. However, results appear inconclusive and are often based on small cross-sectional studies. We aimed to investigate the temporal sequence of the association between screen time and self-esteem taking into account further indirect effects through family or friendship relationship. In our population-based birth cohort study (baseline November 2000–November 2001, Ulm, Germany), these relationships were explored in *n* = 519 11- and 13-year-old children and their parents who both provided information on children’s screen time: time spent watching television or videos (TV), time spent on computers, video game consoles, mobile devices, or cell phones; so called “other screen time”, and children’s self-esteem (KINDL-R). Time watching TV (self-reported) at age 11 was negatively associated with girls’ self-esteem at the same age but positively with an increase of self-esteem between age 11 and 13. However, the latter association was restricted to low to moderate TV viewers. In boys, a higher increase of other screen time between age 11 and age 13 was associated with lower self-reported self-esteem at age 13. Additionally, friendship relationship mediated the association between watching TV and self-esteem in girls. For parental reports similar associations were observed. These findings indicate that time sequence and potential mediators need further investigation in cohort studies with multiple assessments of screen time and self-esteem.

## 1. Introduction

Screen-based sedentary behavior, i.e., behavior characterized by low levels of energy expenditure such as watching television (TV) and computer use, is an increasing public health concern, both in adults and even more in children [1]. In the German “Health Behaviour in School-aged Children” (HBSC) study for example, girls and boys aged 11 to 15 years watched an average of about 2 h of TV on weekdays and 181 (girls) and 202 minutes (boys) on weekend days [2], and these amounts correspond to those reported previously [3,4]. In addition to watching TV, children and preadolescents increasingly use other screen-based devices such as computers, touch-screen phones or tablets, mostly also on a daily basis, adding additional hours of screen time.

Albeit common in children’s life, screen time is associated with an increased risk of cardio-metabolic diseases, overweight, and a large range of physical and psychological disorders in children and adolescents [5,6,7,8,9,10,11]. Additionally, children’s self-esteem may be affected by extensive screen time. Self-esteem reflects the attitude towards the self and may contribute to mental health and social well-being [12]. Following Harter 1999, self-esteem can be captured by a cognitive and a social dimension as well as physical appearance [13]. Each of these aspects may be affected by extensive screen time. Firstly, screen time might be spent at the expense of other activities, like e.g., physical activity, potentially more beneficial for health and cognitive development (displacement hypothesis [14]). Secondly, it can be argued that screen time might be associated with social isolation or poor relationship to friends or family, e.g., [15]. Third, unrealistic body ideals communicated by media might be negatively associated with the physical self-concept and body satisfaction reducing general self-esteem, e.g., [16].

These assumptions concerning a negative effect of screen time on self-esteem are supported—albeit not explicitly tested—by some investigations (see reviews [5,6,7,9,17] and a meta-analysis [18]) but results are not completely conclusive and are often based on small cross-sectional studies unable to enlighten the temporal sequence. The few available intervention studies are primarily targeted at an increase of the activity level and thereby potentially reducing screen time [19,20,21]. Longitudinal studies are, to the best of our knowledge, scarce in this context (apart from [22]). Besides, further covariates considered are mainly restricted to physical activity [20,21,23,24,25], BMI (Body mass index) [8,20,21,25], socioeconomic status [26], race [22,24], or school achievement [27,28]. The role of friends or family relationship, although relevant for the child’s self-esteem [13,29], has rarely been analyzed in recent studies [27,30,31].

Our first aim was to investigate the associations between screen time and self-esteem in children aged 11 and 13 years hypothesizing a negative long-term effect of screen time on self-esteem. A further objective was to explore if family or friendship relationship lay on a causal pathway between screen time and self-esteem, i.e., mediating rather than confounding the effect of screen time on the outcome. In addition, self-reported measures and parent-reported measures of screen time and self-esteem were analyzed separately to reveal potential differences of self- or parental reporting.

## 2. Materials and Methods

### 2.1. Study Description

Data were derived from the Ulm Birth Cohort Study, a birth cohort study with recruitment from the general population in Ulm, West-Germany from November 2000 to November 2001. Response rate at baseline was 67% (1066 of 1593 eligible families, 24 families participated with newborn twins). Details and the primary aim of the study can be found elsewhere [32]. Regular postal follow-ups were conducted including two assessments at 11 and 13 years of a child’s age. At both time points a parental and a child questionnaire were sent to the participants providing a separate sealed envelope for the children. All subjects gave their informed consent for inclusion before they participated in the study. The study was conducted in accordance with the Declaration of Helsinki, and the protocol was approved by the ethics board of Ulm University (No. 98/2000).

### 2.2. Assessment of Self-Esteem

Both child-reported and parent-reported self-esteem were measured separately at ages 11 and 13 years using the KINDL-R self-esteem [33] which covers the past week and consists of 4 items (I was proud of myself, I felt on top of my world, I felt pleased with myself, I had a lot of good ideas, coded as 1 = never to 5 = always). After transformation, the instrument delivers values from 0 to 100 with higher values indicating higher self-esteem. Psychometric analyses revealed moderate internal consistency (Cronbach’s α = 0.78 and 0.79, 11 years and 13 years child-reported and 0.76 and 0.79 parent-reported, respectively)), comparable with previously published data [34].

### 2.3. Screen Time

Information on screen time was reported by children and parents at age 11 years. Both received an own questionnaire covering watching TV or videos at school days and weekend days separately. Respondents were asked how many hours, on average the child watches TV or videos, providing the following categories similar to those used in a large population-based representative study [3]: Not at all, about 30 minutes, 1–2 h, 3–4 h, >4 h/day. The same categories were used to gather information on other screen time: computer, video game console, tablet or cell phone use. To create an index considering weekdays and weekends in one variable, numbers were assigned to the categories (not at all = 0, about 30 minutes = 0.5, 1–2 h = 1.5, 3–4 h = 3.5, >4 h = 5 [3]). These values were weighted, summed up and divided by 7 days ((TV weekday × 5 + TV weekend × 2)/7) indicating the time spent watching TV on an average day. Time spent on computer, video game console, tablet or cell phone use (so called “other screen time”) was handled accordingly.

### 2.4. Possible Confounders

Based on previous literature (details see introduction) the following potential confounders were considered: Child-reported leisure time physical activity (hours/week), children’s BMI based on parent-reported child height and weight (height/weight^2^), maternal educational attainment (<10 years, 10–12 years, >12 years) as proxy for socioeconomic status, maternal nationality (German, other), and the school type attended by the child (secondary general school, intermediate secondary school, grammar school).

### 2.5. Possible Mediators

Satisfaction with friendship (I played with my friends, other kids liked me, I got along well with my friends, I felt different from other children, coded as 1 = never to 5 = always) was regarded as a possible mediator, so was satisfaction with family relationship (I got on well with my parents, I felt fine at home, we quarreled a lot, my parents stopped me doing certain things) derived from KINDL-R. Following the model of Baron and Kenny [35], mediation exists if firstly screen time statistically significant predicts self-esteem, secondly screen time statistically significant predicts family or friendship relationship, and thirdly family or friendship relationship statistically significant predicts self-esteem controlled for screen time.

### 2.6. Statistical Analysis

Baseline characteristics as well as characteristics of the study population are described with 95% confidence interval (CI) to facilitate the comparison. Besides, multivariable adjusted linear regression analyses were conducted using self-esteem as dependent variable, watching TV and other screen time as independent variables. Multicollinearity was excluded as VIF (Variance Inflation Factor) was ≤1.609. We accounted for potential confounders if they were associated with the children’s self-esteem and screen-time at *p* < 0.1 in bivariate sex-stratified regression analyses. According to this definition, maternal educational attainment, maternal nationality, child BMI, and physical activity turned out not to be confounders. There was a significant interaction between watching TV at age 11 years and child’s sex (*p* = 0.028). To check whether satisfaction with family or friendship relationship may be mediators of the association between screen-based sedentary behavior and self-esteem, a Sobel test was performed [36]. This method goes beyond the method of Baron and Kenny [35] as the mediation effect is formally tested. The results of child-reported measurements of children’s screen time and self-esteem are presented in the article. Further results concerning parent-reported measurements are to be found in Table S1 in Supplementary Materials. All statistical analyses were performed with SAS 9.4 (SAS Institute, Cary, NC, USA).

## 3. Results

The final study population consisted of *n* = 519 children, *n* = 246 boys, *n* = 273 girls (among them eight pairs of twins) with an approximately equal distribution of girls and boys (Table 1). 

Compared to the baseline population, the study population included a larger proportion of participants with German nationality and higher maternal education. Average child-reported time watching TV increased from 1.1 h/day (h/d) at age 11 to 1.3 h/d at age 13 years. 

Other screen time (computer, video game consoles, mobile devices, or cell phones) increased from 0.5 h/d at age 11 to 1.5 h/d at age 13, and time spent on physical activity decreased from 6.2 h/week to 6.0 h/week between age 11 and 13. Boys spent more time on other screens than girls, particularly at age 11 (data not shown), and were, at any age, more physically active than their female counterparts. For the respective parent-reported measurements on screen-time and self-esteem see Table S1 in the Supplementary Materials).

Table 2 and Table 3 show considerable variability of self-reported self-esteem over time in children aged 11 to 13 years (Pearson correlation coefficient, (*r* = 0.30 in boys and *r* = 0.23 in girls, each *p* < 0.001)). On the contrary, there was a moderate correlation of self-reported time watching TV (*r* = 0.44 and 0.45, each *p* < 0.001), but low correlation of other screen time between ages 11 and 13 years in girls (*r* = 0.16, *p* = 0.01, boys: *r* = 0.51, *p* < 0.001). Children’s satisfaction with family and friendship relationship was positively correlated with self-esteem at the corresponding age with a small and non-significant association between self-esteem at age 11 and satisfaction with friendship at the same age in boys. There was no statistically significant correlation between BMI and self-esteem. Physical activity at age 13 was significantly correlated with self-esteem at age 11 in boys (*r* = 0.14, *p* = 0.032) but not in girls.

We revealed a negative association between time spent on watching TV at age 11 and the concurrent self-esteem in girls (Table 4, Model 1, unstandardized regression coefficient *b* = −4.03, *p* = 0.017). 

This was also evident when accounting for other screen time (Model 3, *b* = −4.26, *p* = 0.015) and after adjusting for further potential confounders (Model 4). However, no association was seen between watching TV or other screen time at age 11 and self-esteem at age 13. With regard to the change of self-esteem between age 11 and 13, more time spent on watching TV at age 11 was associated with an increase of self-esteem (Model 4, *b* = 5.08, *p* = 0.019, adjusted for school type attended, and satisfaction with family relationship at age 11). Further analyses (data not shown), albeit restricted in power due to a small sample size, revealed that this increase of self-esteem with higher time watching TV at age 11 only holds true in girls watching TV <2 h/d (*b* = 5.78, *p* = 0.071). In the 44 girls watching TV ≥2 h/d a statistically non-significant but strongly negative association was found (*b* = −11.53, *p* = 0.49). Nevertheless, the explained variance of our models ranging from 0 to 8% was rather low (see Table 3).

In boys, we saw no association between watching TV with self-esteem at age 11 as well as with self-esteem at age 13, but other screen time was related to self-esteem. A regression model accounting for watching TV at age 11, other screen time at age 11, and the corresponding changes of screen time between age 13 and age 11 (Table 5, Model 6) revealed that a higher increase of other screen time was associated with lower self-esteem (*b* = −2.93, *p* = 0.034), also evident after adjustment for self-esteem at age 11 (*b* = −2.82, *p* = 0.035).

**Figure 1:**
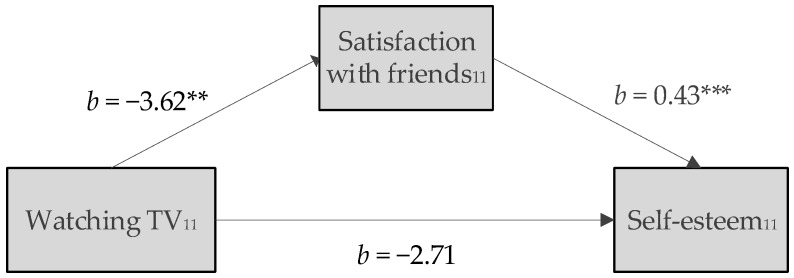
Effect of watching TV at age 11 on self-esteem at age 11 in girls; the negative effect of watching TV is statistically significant mediated by satisfaction with friends; *b* = beta estimate; ** *p* < 0.01, *** *p* < 0.001

In girls, apart from the total effect described, we identified a statistically significant indirect effect of watching TV at age 11 on the concurrent self-esteem mediated by satisfaction with friends (indirect effect (Sobel test) = −1.55, (95% CI −2.77; −0.33)). The direct effect from watching TV at age 11 to self-esteem at age 11 was no longer significant (Figure 1).

The associations between parent-reported time watching TV or other screen-time and parent-reported child’s self-esteem (see Table S2 (girls) and Table S3 (boys) in Supplementary Materials) were similar to the self-reports of the children with stronger long term effects of time watching TV in girls.

## 4. Discussion

To further elicit the relationship between screen time and self-esteem, we examined the association between time watching TV or video and other screen time (computer, video game consoles, mobile devices, or cell phones) at age 11 and self-esteem at age 11 and 13 after considering potential confounders such as physical activity and mediating factors such as relationships to family and friends. Thereby, in girls, watching TV at age 11 was associated with low self-esteem at the same age. Furthermore, we found a significant indirect effect of satisfaction with the relationship to friends mediating the association of screen time on self-esteem in girls. In boys, there was a strong negative association of increase in other screen time between age 11 and 13 on self-esteem at age 13, which persisted after adjustment for potential confounders. Notably, perceptions of children and parents with regard to the association of screen time with their child’s self-esteem were similar but with higher long term effects in the parents’ perception for girls. Physical activity was positively correlated to self-esteem in boys at age 11 but was not shown to be a confounder of the association analyzed.

There are some limitations to be mentioned. Although we accounted for time watching TV and other screen time simultaneously, the observed variables certainly capture only a portion of total sedentary time [37] and may not reflect all possibilities offered by new media, like e.g., the smart phones used for streaming films, etc. However, whether this omnipresence of media leads to a higher impact of other screen time on self-esteem in children is unclear. Furthermore, children’s use of the internet for social purpose (communication with friends, playing online games) or social networking is not considered in details, although it was shown previously that subjects who used social networks more often had poorer self-esteem mediated by greater exposure to social comparisons on social media [38]. Furthermore, the questionnaire we used was not standardized and the categories may not be sensitive to detect small changes of media consumption throughout time. However, the questions corresponded largely to those used in a representative German study [3]. Additionally, although we did not observe large longitudinal effects of media on self-esteem, we did find an association between changes in media consumption and changes of self-esteem. Nevertheless, explained variance of our models is small but accounting for satisfaction with family relationship leads to a rise in explained variance especially in boys, underlining the importance of family relationship in this context. Additionally, we cannot exclude selection bias towards higher educated mothers and mothers with non-German nationality. Media consumption in low socioeconomic status (SES) families is higher than in their high SES counterparts. Thus, also the associations presented might be higher in multiply disadvantaged families as further resources to buffer negative effects of high media exposure might be lacking. A further limitation of the study is that data on screen-time were collected via self-report, as were data on physical activity and BMI. Parent-reports of their children’s BMI are known to be biased [39], a fact which is supported by a lower rate of obesity in our study compared to others (details see review [39]). Most studies observed a negative association of weight, particularly obesity and self-esteem [40] which we could not find. Similar objections may also be raised for the activity level [41,42]. Underreporting of obesity or sedentary behavior would thus lead to an overestimation of the association between screen time and self-esteem which we cannot fully exclude. Nevertheless, there are also some strengths of our study: We observed an indirect effect of friendship relationship mediating the association between watching TV on self-esteem in girls. Although family and friendship relationship are known to play a crucial role in children’s self-esteem throughout development [13,29], family and friendship relationship are not sufficiently considered in recent literature. Furthermore, the longitudinal design and the possibility to also consider parents’ perception of the association between TV time or other screen time and self-esteem are certainly further strengths of our study. The parents’ attitude towards the role of media in their children’s life might be important for interventions often needing both, high motivation of children and parents.

With an average TV time of 1.1 h/d and 1.3 h/d, TV time seems to be lower in our study population whereas time spent on other screens is similar compared to earlier studies [2,3,4], potentially due to a recent increase of computer or internet use. The previously observed sex-difference in screen-based media use (see review [43]) was confirmed. In line with the majority of studies (see reviews [9,17,44]) indicating a negative association between screen time and self-esteem, we could show a harmful effect of time watching TV in girls. However, TV time at age 11 did not predict self-esteem at age 13, potentially indicating a bidirectional relationship between screen-based sedentary behavior and self-esteem. Our results may point towards a higher engagement in TV viewing in girls or more time spent on other screens in boys associated with lower self-esteem. Conversely, it is argued, that the effect of TV viewing on self-esteem may only be short-term [22] which might explain the lacking longitudinal association. However, this assumption seems to be in contrast to some research suggesting that unrealistic body ideals communicated through media might explain the inverse effect of watching TV on self-esteem especially in girls [16]. An adjustment of body ideals might rather be a long lasting process than a short-term change. Furthermore our results do not support the displacement hypothesis, i.e., watching TV or other screen time was not associated with physical activity at any time point in our study.

## 5. Conclusions

Taken together, our study supports a cross-sectional association between watching TV and self-esteem in girls and a similar association between a rise of other screen-time between age 11 and 13 and self-esteem in boys at age 13 but no clear longitudinal effect. From this perspective, besides focusing a potential negative effect of media consumption on self-esteem, parents or physicians may also consider an inverse association. High media consumption may be a sign and not a consequence of low self-esteem. Children who are unsure of their self may spent much time in front of media. From a public health point of view, parents could be sensitized for this aspect to support their children. Certainly, further longitudinal studies are warranted using objectively measured physical activity and BMI to avoid the obstacles of self-reports.

## Figures and Tables

**Table 1:** Characteristics of the study population (*n* = 519) and comparison to baseline population.

Variable	Ulm Birth Cohort StudyBaseline (*n* = 1090 Children)		Study Population (*n* = 519)
	*n*^1^ (%)	(95% CI)	*n*^1^ (%)
**Child’s gender**			
Male	551 (50.6)	(47.7; 53.6)	246 (47.4)
Female	537 (49.4)	(46.4; 52.3)	273 (52.6)
**Maternal nationality**			
German	930 (85.6)	(83.5; 87.6)	480 (92.5)
Other	157 (14.4)	(12.4; 16.5)	39 (7.5)
**Maternal education**			
< 10 years	252 (23.8)	(21.2; 26.3)	62 (12.0)
10–12 years	410 (38.7)	(35.7; 41.6)	203 (39.4)
> 12 years	398 (37.5)	(34.6; 40.5)	250 (48.5)
**Single parenthood** at age 11			
Yes	60 (9.9)	(7.5; 12.2)	47 (9.1)
No	548 (90.1)	(87.8; 92.5)	469 (90.9)
**Child’s BMI** at age 11, parent-reported			
Underweight (< P10)			60 (13.1)
Normal (P10–P90)			367 (80.0)
Overweight (> P90)			32 (7.0)
**Self-esteem** at age 11, mean (SD), child-reported			63.5 (18.1)
**Self-esteem** at age 13, mean (SD), child-reported			60.7 (17.4)
**Difference of self-esteem** at age 13 and 11, mean (SD), child-reported			
Male			−1.1 (22.2)
Female			−4.3 (20.8)
**Watching TV** at age 11 (h/d), mean (SD), child-reported			1.1 (0.7)
**Watching TV** at age 13 (h/d), mean (SD), child-reported			1.3 (0.8)
**Other screen time** at age 11 (h/d), mean (SD), child-reported			0.5 (0.5)
**Other screen time** at age 13 (h/d), mean (SD), child-reported			1.5 (1.2)
**Physical activity** at age 11 (h/w), mean (SD), child-reported			6.2 (3.9)
**Physical activity** at age 13 (h/w), mean (SD), child-reported			6.0 (4.2)
**Satisfaction with family relationship** at age 11, mean (SD), child-reported			87.1 (11.3)
**Satisfaction with family relationship** at age 13, mean (SD), child-reported			86.2 (13.2)
**Satisfaction with friendship relationship** at age 11, mean (SD), child-reported			80.1 (15.0)
**Satisfaction with friendship relationship** at age 13, mean (SD), child-reported			79.9 (14.5)

^1^ sum may not add up to total because of missing values for some variables; Abbreviations: CI: Confidence intervals, SD: Standard deviation, P: Percentile, h/d: hours/day; h/w: hours/week.

**Table 2:** Pearson correlation coefficients between study variables in boys.^1^

		1	2	3	4	5	6	7	8	9	10	11	12	13	14
1	Self-esteem_11_	-	0.30 ***	0.03	0.01	−0.09	−0.05	0.04	0.14 *	0.36 ***	0.15 *	0.31 ***	0.09	0.10	0.02
2	Self-esteem_13_		-	0.07	−0.06	−0.10	−0.16 *	0.10	0.04	0.22 ***	0.25 ***	0.28 ***	0.38 ***	0.00	0.08
3	Watching TV_11_			-	0.44 ***	0.32 ***	0.21 ***	−0.05	−0.02	−0.03	−0.00	0.04	0.07	0.11	0.10
4	Watching TV_13_				-	0.16 *	0.25 ***	−0.04	0.06	−0.11	−0.07	−0.04	−0.05	0.21 ***	0.28 ***
5	Other screen time_11_					-	0.51 ***	−0.04	−0.08	−0.12	−0.15 **	−0.11	−0.10	−0.05	−0.03
6	Other screen time_13_						-	−0.13	−0.04	−0.02	−0.12	0.05	−0.10	0.00	0.04
7	Physical activity_11_							-	0.30 ***	−0.00	0.14 *	0.14 *	0.18 **	−0.08	−0.07
8	Physical activity_13_								-	0.10	0.09	0.05	0.14 *	−0.06	−0.01
9	Satisfaction with family relationship_11_									-	0.37 ***	0.33 ***	0.23 ***	−0.11	−0.11 *
10	Satisfaction with family relationship_13_										-	0.34 ***	0.36 ***	0.08	0.06
11	Satisfaction with friends relationship_11_											-	0.41 ***	0.01	0.07
12	Satisfaction with friends relationship_13_												-	0.00	0.04
13	BMI_11_													-	0.85 ***
14	BMI_13_														-

* p < 0.05, ** *p* < 0.01, *** *p* < 0.001; Abbreviations: TV: Television, BMI: Body mass index; ^1^ the subscript numbers indicate the age of the children (11 or 13 years); respective row labels also identify corresponding column header; all variables other than BMI are child-reported.

**Table 3:** Pearson correlation coefficients between study variables in girls.^1^

		1	2	3	4	5	6	7	8	9	10	11	12	13	14
1	Self-esteem_11_	-	0.23 ***	−0.16 *	−0.05	−0.02	−0.04	0.06	−0.03	0.16 *	0.09	0.34 ***	0.19 **	−0.10	−0.09
2	Self-esteem_13_		-	−0.01	0.03	−0.01	−0.07	0.05	0.02	0.12 *	0.33 ***	0.16 ***	0.33 ***	−0.02	0.01
3	Watching TV_11_			-	0.45 ***	0.22 ***	0.24 ***	−0.12	−0.03	−0.18 **	0.00	−0.17 **	−0.10	0.17 **	0.13 *
4	Watching TV_13_				-	0.13 *	0.31 ***	−0.10	0.02	−0.04	0.04	−0.05	0.05	0.10	0.09
5	Other screen time_11_					-	0.16 ***	−0.11	−0.02	−0.11	0.00	−0.06	−0.13	0.11	0.12
6	Other screen time_13_						-	0.00	−0.05	0.03	−0.06	0.05	0.11	0.09	0.10
7	Physical activity_11_							-	0.32 ***	−0.01	0.29	0.08	0.14 *	0.02	0.06
8	Physical activity_13_								-	−0.09	−0.09	0.06	0.07	−0.05	−0.04
9	Satisfaction with family relationship_11_									-	0.19 **	0.28 ***	0.16 **	−0.03	−0.08
10	Satisfaction with family relationship_13_										-	0.17 **	0.31 ***	0.04	−0.00
11	Satisfaction with friends relationship_11_											-	0.47 ***	−0.13 *	−0.14 *
12	Satisfaction with friends relationship_13_												-	−0.05	−0.05
13	BMI_11_													-	0.83 ***
14	BMI_13_														-

* p < 0.05, ** *p* < 0.01, *** *p* < 0.001; Abbreviations: TV: Television, BMI: Body mass index; ^1^ the subscript numbers indicate the age of the children (11 or 13 years); respective row labels also identify corresponding column header; all variables other than BMI are child-reported.

**Table 4:** Determinants of self-esteem in girls (*n* = 273), all variables are child-reported. ^1^

	Self-Esteem_11_			Self-Esteem_13_			Self-Esteem_13-11_		
	*b*	*p*	R^2^	*b*	*p*	R^2^	*b*	*p*	R^2^
**Model 1: crude**									
Watching TV_11_ (h/d)	**−4.03**	**0.017**	0.02	−0.42	0.79	0.00	3.61	0.071	0.01
**Model 2: crude**									
Other screen time11 (h/d)	−0.83	0.75	0.00	−0.35	0.88	0.00	0.48	0.87	0.01
**Model 3: mutually adjusted**									
Watching TV11 (h/d)	**−4.26**	**0.015**	0.02	−0.44	0.78	0.00	3.82	0.067	0.01
Other screen time11 (h/d)	0.40	0.88		−0.28	0.91		−0.69	0.83	
**Model 4 ^2^**									
Watching TV11 (h/d)	**−3.96**	**0.034**	0.04	−0.15	0.79	0.13	**5.08**	**0.019**	0.04
Other screen time11 (h/d)	1.38	0.62		−0.84	0.54		−1.55	0.54	
**Model 5 ^3^**									
Watching TV11 (h/d)				2.20	0.25	0.04	**7.06**	**0.005**	0.05
Other screen time11 (h/d)				−1.37	0.61		−2.92	0.41	
TV13 – TV11 (h/d)				1.85	0.21		2.69	0.17	
Other screen time13 - other screen time11 (h/d)				−0.89	0.30		−1.94	0.085	
**Model 6 ^4^**									
Watching TV11 (h/d)				3.18	0.093	0.09			
Other screen time11 (h/d)				−1.68	0.52				
TV13 – TV11 (h/d)				2.02	0.17				
Other screen time13 - other screen time11 (h/d)				−1.10	0.19				

Abbreviations: *b*: beta estimates; bold letters indicate statistical significance at *p* < 0.05; h/d: hours/day; ^1^ unstandardized coefficients are presented. The subscript numbers indicate the age of the children (11 or 13 years); ^2^ further adjusted for school type, and satisfaction with family relationship_11_ (self-esteem_11_ and self-esteem_13-11_)_,_ or family relationship_13_ (self-esteem_13_); ^3^ further adjusted for school type, satisfaction with family relationship_13_, time spent on watching TV_13_ + other screen time_13_; ^4^ further adjusted for school type, satisfaction with family relationship_13_, time spent on watching TV_13_ + other screen time_13_, and self-esteem_11._

**Table 5:** Determinants of child-reported self-esteem in boys (*n* = 246), all variables are child-reported. ^1.^

	Self-Esteem_11_			Self-Esteem_13_			Self-Esteem_13-11_		
	*b*	*p*	R^2^	*b*	*p*	R^2^	*b*	*p*	R^2^
**Model 1: crude**									
Watching TV_11_ (h/d)	0.77	0.62	0.00	1.71	0.28	0.00	0.94	0.61	0.00
**Model 2: crude**									
Other screen time_11_ (h/d)	−2.95	0.19	0.01	−3.38	0.13	0.01	−0.43	0.87	0.00
**Model 3: mutually adjusted**									
Watching TV_11_ (h/d)	1.49	0.39	0.01	2.13	0.21	0.02	0.64	0.75	0.00
Other screen time_11_ (h/d)	−3.63	0.12		−4.31	0.066		−0.68	0.81	
**Model 4 ^2^**									
Watching TV_11_ (h/d)	1.42	0.39	0.13	1.72	0.18	0.08	0.57	0.78	0.01
Other screen time_11_ (h/d)	−2.12	0.34		−2.56	0.25		0.05	0.99	
**Model 5 ^3^**									
Watching TV_11_ (h/d)				2.47	0.22	0.10	0.67	0.78	0.03
Other screen time_11_ (h/d)				−4.09	0.075		−2.09	0.46	
TV_13 –_ TV_11_ (h/d)				−0.73	0.68		−1.61	0.45	
Other screen time_13_ - other screen time_11_ (h/d)				**−2.93**	**0.034**		−2.51	0.14	
**Model 6 ^4^**									
Watching TV_11_ (h/d)				2.02	0.30	0.16			
Other screen time_11_ (h/d)				−3.59	0.11				
TV_13 –_ TV_11_ (h/d)				−0.96	0.58				
Other screen time_13_ - other screen time_11_ (h/d)				**−2.82**	**0.035**				

Abbreviations: *b*: beta estimates; bold letters indicate statistical significance at *p* < 0.05; h/d: hours/day; ^1^ unstandardized coefficients are presented. The subscript numbers indicate the age of the children (11 or 13 years); ^2^ further adjusted for school type, and satisfaction with family relationship_11_ or relationship_13_ depending on the outcome; ^3^ further adjusted for school type, satisfaction with family relationship_13_, time spent on watching TV_13_ + other screen time_13_; ^4^ further adjusted for school type, satisfaction with family relationship_13_, time spent on watching TV_13_ + other screen time_13_, and self-esteem_11._

## Supplementary Materials

The following are available online at http://www.mdpi.com/1660-4601/15/6/1275/s1. Table S1: Characteristics of study-population, parent-reported variables, Table S2: Determinants of parent-reported self-esteem in girls, all variables are parent-reported (*n* = 274), Table S3. Determinants of parent-reported self-esteem in boys, all variables are parent-reported (*n* = 246).
