# Supplementary material for: Screen Time, Physical Activity and Self-Esteem in Children: The Ulm Birth Cohort Study
Source: Int J Environ Res Public Health. 2018 Jun 16;15(6):1275. doi: 10.3390/ijerph15061275 (PMC6025387; doi:10.3390/ijerph15061275)
Supplement: Supplementary file 1 [file ijerph-15-01275-s001.pdf]

Supplementary Materials

Table S1. Characteristics of study-population, parent-reported variables

**Variable**	**Study population (n = 520)** [1]
	**Mean, SD**
**Self-esteem** child age 11, mean (SD), parent-reported	71.1 (12.4)
**Self-esteem** child age 13, mean (SD), parent-reported	69.5 (14.0)
Difference of **self-esteem** child age 13 and 11, mean (SD), parent -reported	
Male	-1.4 (14.5)
Female	-2.2 (15.0)
**Watching TV** child age 11 (h/d), mean (SD), parent-reported	1.1 (0.7)
**Watching TV** child age 13 (h/d), mean (SD), parent-reported	1.4 (0.7)
**Other screen time** child age 11 (h/d), mean (SD), parent-reported	0.6 (0.5)
**Other screen time** child age 13 (h/d), mean (SD), parent-reported	2.8 (1.1)
**Satisfaction with family relationship** child age 11, mean (SD), parent-reported	79.8 (12.4)
**Satisfaction with family relationship** child age 13, mean (SD), parent-reported	78.5 (13.2)
**Satisfaction with friendship relationship** child age 11, mean (SD), parent-reported	77.2 (13.2)
**Satisfaction with friendship relationship** child age 13, mean (SD), parent-reported	76.7 (14.1)

[1] total n may differ because of missing values for some variables

Table S2. Determinants of parent-reported self-esteem in boys (n = 246), all variables are parent-reported.[1]

	$Self\text{-}esteem_{11}$			$Self\text{-}esteem_{13}$			$Self\text{-}esteem_{13\text{-}11}$		
	**b**	**p**	R^2	**b**	**p**	R^2	**b**	**p**	R^2
**Model 1: crude**									
Watching TV_{11} (h/d)	-0.32	0.79	0.00	1.31	0.29	0.00	1.62	0.21	0.01
**Model 2: crude**									
Other screen $time_{11}$ (h/d)	-1.61	0.31	0.00	-0.60	0.72	0.00	1.01	0.57	0.00
**Model 3: mutually adjusted**									
Watching TV_{11} (h/d)	0.29	0.82	0.00	2.24	0.09	0.00	1.95	0.17	0.00
Other screen $time_{11}$ (h/d)	-1.75	0.31		-1.70	0.34		0.05	0.99	
**Model 4** [2]									
Watching TV_{11} (h/d)	-0.04	0.97	0.19	1.89	0.16	0.05	1.93	0.17	0.06
Other screen $time_{11}$ (h/d)	-1.83	0.25		-1.95	0.28		-0.11	0.95	
**Model 5** [3]									
Watching TV_{11} (h/d)				1.72	0.27		1.88	0.26	
Other screen $time_{11}$ (h/d)				-2.76	0.19		-0.23	0.92	
TV_{13} - TV_{11} (h/d)				-2.29	0.74	0.05	-2.72	0.10	0.11
Other screen $time_{13}$ - other screen $time_{11}$ (h/d)				-0.34	0.15		0.30	0.78	
**Model 6** [4]									
Watching TV_{11} (h/d)				1.76	0.174				
Other screen $time_{11}$ (h/d)				-1.63	0.36				
TV_{13} - TV_{11} (h/d)				-2.48	0.093	0.20			
Other screen $time_{13}$ - other screen $time_{11}$ (h/d)				-0.05	0.95				

Abbreviations: b: beta estimates; h/d: hours/day

[1] unstandardized coefficients are presented. The subscript numbers indicate the age of the children (11 or 13 years)

[2] further adjusted for school type, and satisfaction with family $relationship_{11}$ or $relationship_{13}$ depending on the outcome

[3] further adjusted for school type, satisfaction with family $relationship_{13}$, time spent on watching TV_{13} + other screen $time_{13}$

[4] further adjusted for school type, satisfaction with family $relationship_{13}$, time spent on watching TV_{13} + other screen $time_{13}$, and $self\text{-}esteem_{11}$

Table S3. Determinants of parent-reported self-esteem in girls (n = 274), all variables are parent-reported. [1]

	Self-esteem$_{11}$			Self-esteem$_{13}$			Self-esteem$_{13-11}$		
	b	p	R^2	b	p	R^2	b	p	R^2
**Model 1: crude**									
Watching TV$_{11}$ (h/d)	-4.06	**<0.001**	0.05	-3.95	**0.004**	0.03	0.00	1.00	0.00
**Model 2: crude**									
Other screen time$_{11}$ (h/d)	-3.64	**0.027**	0.02	-4.38	**0.034**	0.02	-0.82	0.70	0.00
**Model 3: mutually adjusted**									
Watching TV$_{11}$ (h/d)	-3.61	**0.002**	0.05	-3.45	**0.015**	0.04	0.07	0.96	0.00
Other screen time$_{11}$ (h/d)	-2.03	0.25		-2.68	0.21		-0.72	0.75	
**Model 4** [2]									
Watching TV$_{11}$ (h/d)	-2.65	**0.022**	0.19	-3.71	**0.014**	0.05	-1.05	0.50	0.03
Other screen time$_{11}$ (h/d)	-2.49	0.14		-2.19	0.32		0.21	0.93	
**Model 5** [3]									
Watching TV$_{11}$ (h/d)				-2.95	0.095		-0.25	0.89	
Other screen time$_{11}$ (h/d)				-3.41	0.14		-0.51	0.83	
TV$_{13}$ – TV$_{11}$ (h/d)				0.13	0.21	0.07	-0.13	0.94	0.04
Other screen time$_{13}$ - other screen time$_{11}$ (h/d)				-1.16	0.94		-0.44	0.63	
**Model 6** [4]									
Watching TV$_{11}$ (h/d)				-1.62	0.32				
Other screen time$_{11}$ (h/d)				-1.99	0.36				
TV$_{13}$ – TV$_{11}$ (h/d)				0.00	1.00	0.18			
Other screen time$_{13}$ - other screen time$_{11}$ (h/d)				-0.80	0.35				

Abbreviations: b: beta estimates; bold letters indicate statistical significance at $p < 0.05$; h/d: hours/day

[1] unstandardized coefficients are presented. The subscript numbers indicate the age of the children (11 or 13 years)

[2] further adjusted for school type, and satisfaction with family relationship$_{11}$ or relationship$_{13}$ depending on the outcome

[3] further adjusted for school type, satisfaction with family relationship$_{13}$, time spent on watching TV$_{13}$ + other screen time$_{13}$

[4] further adjusted for school type, satisfaction with family relationship$_{13}$, time spent on watching TV$_{13}$ + other screen time$_{13}$, and self-esteem$_{11}$
